# A Novel Sarcopenia Screening Score Based on Thyroid Function Parameters in Euthyroid Middle‐Aged and Elderly Chinese Adults

**DOI:** 10.1155/ije/1577695

**Published:** 2026-06-24

**Authors:** Fei Ding, Jirong Yue, Yong He

**Affiliations:** ^1^ Department of Laboratory Medicine, West China Hospital, Sichuan University, Chengdu, Sichuan, China, scu.edu.cn; ^2^ Department of Laboratory Medicine, Clinical Laboratory Medicine Research Center Chengdu, Chengdu, Sichuan, China, scu.edu.cn; ^3^ West China Hospital, Sichuan Clinical Research Center for Laboratory Medicine Chengdu, Sichuan University, Chengdu, Sichuan, China, scu.edu.cn; ^4^ Department of Geriatrics and National Clinical Research Center for Geriatrics, West China Hospital, Sichuan University, Chengdu, Sichuan, China, scu.edu.cn

**Keywords:** FT4, LASSO regression, middle-aged and elderly participants, sarcopenia, screening score, thyroid hormone

## Abstract

**Background:**

This research aims to develop an alternative sarcopenia screening score based on thyroid hormone function parameters, intended for screening and validating the probability of having sarcopenia among middle‐aged and elderly participants with normal thyroid function tests in China.

**Methods:**

This multicenter, cross‐sectional study assessed the prevalence of sarcopenia among 3,858 thyroid‐healthy Chinese adults across hospitals in western China hospitals. Participants were randomized to exploratory and validation cohorts. After applying Lasso to select variables, multivariate logistic regression analysis was conducted. We developed a novel sarcopenia screening score from exploratory data, subsequently validating it internally across both cohorts.

**Results:**

The new sarcopenia screening score comprises five variables: age, body mass index (BMI), calf circumference (CC), aspartate aminotransferase/alanine aminotransferase ratio (AST/ALT), and free T4 (FT4), with a scoring range of 0–14. The area under the receiver operating characteristic (ROC) curve for screening for sarcopenia was 0.845 (95% CI: 0.831–0.859) in the exploratory group and 0.854 (95% CI: 0.831–0.874) in the validation group. At the optimal cutoff of 3, the sensitivity and specificity were 78.3% and 75.1%, respectively, in the exploratory cohort; in the validation cohort, with a cutoff of 4, sensitivity and specificity were 68.4% and 87.0%. The Brier score was 0.092 in the exploratory cohort and 0.068 in the validation cohort, confirming satisfactory calibration.

**Conclusion:**

The newly developed sarcopenia screening score shows promise as a practical tool for identifying sarcopenia in middle‐aged and elderly participants with normal thyroid function tests in China.

## 1. Introduction

Sarcopenia, essentially a loss of muscle strength or fitness linked to the aging process, is a key factor that can result in disability and even increased mortality rates [1]. It is now widely recognized as a new category in geriatric medicine, acting as a pivotal step toward a more advanced state of frailty in the elderly individuals [2]. The development of sarcopenia is a complex process involving several mechanisms, including issues with insulin signaling, inflammation, and fluctuations in hormone levels—all of which worsen sarcopenia [3]. Key hormones such as insulin, testosterone, growth hormone, and vitamin D (vit D) are pivotal in driving the progression of sarcopenia. Fortunately, it is not a lost cause; sarcopenia can be treated and prevented [1, 4]. It has been noted that stepping in early, when someone is still physically capable, is more effective than waiting until the situation is more dire [5]. This makes the early stages of sarcopenia a prime time for intervention to mitigate its progression and prevent disability [6, 7]. However, surprisingly, sarcopenia often occurs under the radar and is treated poorly in conventional medicine. This is largely because there is a lack of clarity on how to measure and diagnose it effectively, as well as assess treatment success. Typically, people with sarcopenia only realize their condition when the decline in muscle mass and function becomes quite severe, leading to significant disabilities. Thus, incorporating regular health checks before physical issues arise could be the key to catching sarcopenia early and making a real difference.

The Asian Working Group for Sarcopenia (AWGS) [8] recommends diagnostic criteria for sarcopenia that require either low muscle strength or poor physical performance, in addition to reduced muscle mass. Typically, muscle strength is assessed through grip strength, whereas physical performance is evaluated using short physical performance battery tests or usual gait speed. Muscle mass, on the other hand, is estimated using bioelectrical impedance analysis (BIA) or dual‐energy X‐ray absorptiometry (DXA). The diagnosis of sarcopenia relies on specialized equipment and is time‐consuming and often constrained by the need for specialized tools and training in many clinical settings. Consequently, there is a pressing need for a concise, alternative screening tool for sarcopenia that does not depend on numerous instruments. The recently released AWGS 2025 [9] consensus update has shifted the diagnostic emphasis toward muscle health and early functional decline, recommending screening even in middle‐aged adults without overt functional impairment. The SARC‐F [10] questionnaire has been utilized for large‐scale population screening in epidemiological studies, but it has drawn criticism because of its low to moderate sensitivity, which can lead to false‐negative results in sarcopenia screening, making it less than ideal for this purpose.

In recent years, research has revealed that thyroid hormones play a crucial role in muscle physiology through mitochondrial function and glucose metabolism [11]. Fluctuations in thyroid hormone levels have been linked to sarcopenia, even among individuals without overt thyroid dysfunction [12]. The thyroid hormone sensitivity index (THSI), encompassing measures such as the free T3 (FT3) to free T4 (FT4) ratio (FT3/FT4), T4 resistance index of thyroid‐stimulating hormone (TSH) (TT4RI), thyroid‐stimulating hormone index (TSHI), T3 resistance index of TSH (TT3RI), free T3 index based on thyroid feedback quartiles (TFQIFT3), and TFQIFT4, stands out as a sensitive barometer of thyroid metabolic function [13–15]. However, to the best of our knowledge, existing studies have focused primarily on examining the relationship between thyroid hormones and sarcopenia and have not translated these findings into practical screening applications for high‐risk populations.

Catching sarcopenia early is crucial as it provides opportunities for interventions, particularly when it is diagnosed at an early stage. Screening tools can play a pivotal role in identifying the condition, paving the way for effective treatment, especially among those at high risk. For these reasons, a straightforward, thyroid hormone–based clinical screening tool for sarcopenia that is easy to implement in routine practice is urgently needed. To that end, we designed a study aimed at developing a simple sarcopenia screening tool and evaluating its ability to estimate the probability of sarcopenia on the basis of a multicenter, cross‐sectional investigation. This study sought to determine the prevalence of sarcopenia among older adults with normal thyroid function and was conducted across several hospitals in western China. Our study included a total of 3,858 participants who were randomly divided into two groups: an exploratory cohort and a validation cohort. Multivariate logistic regression analysis of the exploratory cohort revealed a sarcopenia screening score to more accurately determine the probability of having sarcopenia in individuals with normal thyroid function. This screening score was subsequently validated in the verification cohort to assess its suitability as a novel alternative screening tool for detecting sarcopenia in middle‐aged and elderly Chinese individuals with normal thyroid function.

## 2. Materials and Methods

### 2.1. Study Sample

Utilizing baseline data from the 2018 WCHAT prospective cohort study, this analysis was initiated. This effort was a collaborative feat involving seven medical centers, with key participants including Sichuan University’s West China Hospital, Xinjiang Medical University’s Fifth Hospital, Chengdu’s Fifth and Eighth People’s Hospitals, Yan’an Affiliated Hospital, the Guizhou Province People’s Hospital, and the First Affiliated Hospital of Kunming Medical University. Qualified participants for the research project were people over the age of fifty who had happily given their informed consent, had been living in the area for no less than three years, were not hindered by communication issues, and could endure a full 50‐min interview session. Individuals were excluded from the study if they (1) had suffered from serious ailments such as severe respiratory distress or acute conditions affecting the heart, kidneys, or liver; (2) had a projected lifespan of less than 6 months; (3) had previously experienced thyroid problems and thyroid disorders or had undergone antithyroid therapy, and patients were currently receiving hormone replacement treatments (including corticosteroids or thyroid hormones), or anyone who was taking medications known to influence thyroid activity (for example, amiodarone); (4) were unable to autonomously perform the required physical examination tasks; or (5) failed to show interest in taking part in the research endeavor. We had to exclude a few patients (Supporting Figure 1)—3036 did not receive sarcopenia evaluations, another 401 had gaps in their data or laboratory tests, and 241 individuals with thyroid dysfunction were excluded. With these exclusions accounted for, a total of 3,858 individuals aged 50 and older who satisfied the aforementioned criteria were included in our current data analysis. The participants were randomly split into two distinct cohorts: the exploratory group and the validation group. To develop a novel risk assessment tool for sarcopenia and conduct internal validation, multivariate logistic regression analysis was performed on the exploratory group. Moreover, the validation group underwent internal reliability assessments to ensure the robustness of our findings. The participant selection flowchart is shown in Supporting Figure 1.

### 2.2. Assessment of Sarcopenia

In this investigation, researchers relied on the AWGS 2019 diagnostic criteria [9] to identify cases of sarcopenia given their widespread adoption throughout Asian medical communities. These guidelines take into account the trifecta of muscle depletion, diminished strength, and compromised physical function. To assess muscle strength, participants were asked to squeeze a handheld dynamometer (EH101; Camry, Zhongshan, China) twice with their dominant hand, and the superior reading was selected for data analysis. The threshold for low muscle mass was established at an appendicular skeletal muscle mass index (SMI) below 7.0 kg/m^2^ for male subjects and under 5.7 kg/m^2^ for females, as calculated via bioimpedance analysis. Furthermore, according to the AWGS protocol, handgrip strength measurements falling short of 28 kg for men and 18 kg for women were also considered indicative of diminished strength. Physical performance was evaluated through the recommended 6‐m walk test, with speeds crawling below 1.0 m/s raising red flags for potential sarcopenia patients.

### 2.3. Additional Measurements

Participant age, gender, alcohol consumption, and current smoking‐related information were collected. The height and weight of the study participants were measured by trained personnel using a standardized approach with a CSTF—5,000 style apparatus (Tongfang Health Technology Co., Ltd.). Researchers employed a nonstretchable flexible measuring tape to determine the CC in a controlled, peaceful environment where participants maintained their knees and ankles at right angles. By adjusting the tape as needed, the team identified the widest horizontal circumference around the calf while being careful to avoid any compression of the skin during the measurement process. In addition, systolic blood pressure (SBP) and diastolic blood pressure (DBP) were also measured.

The subjects abstained from food for a minimum of 10 h preceding blood withdrawal. The fasting blood glucose (GLU), triglyceride (TG), total cholesterol (CHOL), high‐density lipoprotein (HDL), low‐density lipoprotein (LDL), estimated glomerular filtration rate (eGFR), uric acid (UA), AST, and ALT concentrations were measured with an Olympus AU400 instrument from Japan. Thyroid hormones (TSH, FT3, and FT4) and fasting insulin (INS) were quantified with a German Roche cobase 411 automated immunoanalyzer. Vit D levels were ascertained with an Abbott i20005R immunoanalyzer, an instrument from the United States.

### 2.4. Computational Formula

Body mass index (BMI) was calculated as weight divided by height squared; Homeostatic model assessment for insulin resistance (HOMA‐IR) was calculated by the formula: HOMAIR=(INS×GLU)/22.5, where INS represented fasting insulin and GLU represented fasting glucose. The AST/ALT ratio was calculated by dividing AST level by ALT level. Thyroid hormone sensitivity indices were determined by calculating the FT3/FT4 ratio, which revealed that peripheral thyroid hormone sensitivity is heightened if the FT3/FT4 ratio is elevated. The TT4RI and TT3RI formulas are used—TT4RI equals FT4 multiplied by TSH and TT3RI equals FT3 multiplied by TSH. Central thyroid hormone sensitivity is inversely proportional to TT4RI and TSHI values. For TFQIFT4, subtract (1‐cdfTSH) from the cumulative distribution function (CDF) of FT4, and for TFQIFT3, do the same with FT3. The TFQI coefficient is derived from the medical examination population. Unlike TT4RI and TSHI, TFQI is preferable because it does not produce outliers, even during periods of thyroid dysfunction. The TFQI ranges from −1 to 1; a positive value indicates normal insensitivity, whereas a negative value suggests that the hypothalamic–pituitary–thyroid (HPT) axis is more sensitive to changes in FT4/FT3.

### 2.5. Statistical Analyses

SPSS 20 was the software of choice for our statistical analysis, hailing from the city of Windy, Chicago, Illinois. We subjected all the parameters to a normality test. Continuous data adhering to a normal distribution are reported as the means with their respective standard deviations, whereas categorical data are presented as counts and percentages. To differentiate between groups, we employed an independent samples *t*‐test for means and a chi‐square test for percentages. Data with a nonnormal distribution are presented as medians with interquartile ranges and were evaluated through nonparametric methods. We employed restricted cubic spline analysis to delve into the nonlinear interplay between variables and the incidence of sarcopenia. To minimize overfitting and selection bias, we performed variable selection using least absolute shrinkage and selection operator (LASSO) regression with 10‐fold cross‐validation. The optimal regularization parameter (*λ*) was chosen according to the 1‐standard error (1‐SE) rule, which yields the most parsimonious model with performance within one standard error of the minimum. Candidate variables entered into LASSO included age, gender, BMI, calf circumference (CC), AST/ALT ratio, FT3, FT4, FT3/FT4 ratio, TT3RI, TT4RI, TSHI, TFQIFT3, TFQIFT4, INS, HOMA‐IR, fasting glucose, TGs, HDL, LDL, creatinine, eGFR, uric acid, vit D, current drinking, and current smoking status. LASSO selected five variables with nonzero coefficients at *λ* = 0.0167 (lambda.1se): FT4, AST/ALT ratio, age, CC, and BMI. These five variables were subsequently entered into a multivariable logistic regression model to construct the sarcopenia screening score. We developed a sarcopenia screening score by the regression coefficients of significant variables, making it more practical for clinical use. A *p* value above 0.05 was considered to indicate statistical nonsignificance. The sarcopenia screening score, initially developed in an exploratory group, was subsequently validated in a separate population. With MedCalc 20 at our disposal, we utilized the receiver operating characteristic (ROC) curve area under the curve (AUC) to evaluate the discriminatory power of the model in ranking participants on the basis of the likelihood of muscle wasting. The C‐statistic was computed to compare the AUCs. ROC curves were plotted by plotting sensitivity against 1‐specificity for each gender‐stratified cutoff value. The Youden’s index, which maximizes the sum of sensitivity and specificity minus one, was used to pinpoint the optimal cutoff point. We calculated the positive and negative predictive values at this threshold. The discriminative ability of the score was assessed by the AUC. Calibration was evaluated using calibration plots (observed vs. predicted probabilities) and the Brier score. A Brier score < 0.25 indicates satisfactory calibration.

## 3. Results

### 3.1. Participant Demographics and Sarcopenia Occurrence Rates

Table 1 outlines the initial traits of individuals in both the exploratory and validation groups, categorizing them on the basis of whether they had sarcopenia. The exploratory group included 2,768 euthyroid individuals aged 50 years and older, with 2,261 serving as the control group without sarcopenia and 507 serving as the sarcopenia group.

**TABLE 1:** Baseline characteristics of participants in the exploratory subjects and validation subjects with or without sarcopenia.

Variables	Exploratory subjects			Validation subjects		
	Control group (*n* = 2261)	Sarcopenia (*n* = 507)	*p* value	Control group (*n* = 973)	Sarcopenia (*n* = 117)	*p* value
Age (years)	60 (54, 66)	66 (60,73)	< 0.001	62 (55, 66)	68 (60, 74)	< 0.001
Male, *n* (%)	841 (37.2)	212 (41.8)	0.055	499 (51.3)	56 (47.9)	0.274
SBP (mmHg)	128 (115,141)	126 (113, 139)	0.068	129 (116, 141)	130 (117, 143)	0.784
DBP (mmHg)	81 (74, 89)	78 (71, 87)	< 0.001	82 (74, 89)	79 (72, 86)	0.011
Hypertension, *n* (%)	646 (28.6)	127 (25.0)	0.113	293 (30.1)	35 (29.9)	0.529
Diabetes, *n* (%)	178 (7.9)	43 (8.5)	0.651	78 (8.0)	11 (9.4)	0.355
Dyslipidemia, *n* (%)	478 (21.1)	62 (12.2)	< 0.001	187 (19.2)	11 (9.4)	0.004
Current smoking, *n* (%)	401 (17.7)	116 (22.9)	0.008	386 (39.7)	47 (40.2)	0.496
Drinking, *n* (%)	631 (27.9)	129 (25.4)	0.271	441 (45.3)	47 (40.2)	0.168
BMI (kg/m^2^)	25.74 (23.52, 28.13)	21.89 (20.10, 23.68)	< 0.001	25.94 (23.71, 28.24)	21.90 (20.08, 23.69)	< 0.001
TSH (mIU/L)	2.32 (1.65, 3.08)	2.18 (1.56, 2.97)	0.05	2.37 (1.68, 3.10)	2.46 (1.75, 3.10)	0.926
FT3 (pmol/L)	4.57 (4.21, 4.98)	4.44 (4.09, 4.87)	< 0.001	4.54 (4.18, 4.97)	4.42 (4.14, 4.76)	0.015
FT4 (pmol/L)	18.08 (16.53, 19.64)	18.61 (16.79, 20.36)	< 0.001	18.44 (17.12, 19.87)	18.93 (17.55, 20.22)	0.031
FT3/FT4	0.25 (0.23, 0.28)	0.24 (0.22, 0.27)	< 0.001	0.25 (0.23, 0.27)	0.24 (0.22, 0.25)	< 0.001
TSHI	3.25 (2.91, 3.57)	3.28 (2.90, 3.61)	0.387	3.33 (3.00, 3.62)	3.42 (3.05, 3.70)	0.188
TT3RI	10.68 (7.53, 13.99)	9.79 (7.06, 13.20)	0.003	11.03 (7.63, 14.18)	10.53 (7.93, 13.40)	0.479
TT4RI	41.57 (29.86, 54.63)	41.32 (28.60, 54.54)	0.452	43.45 (31.40, 56.44)	45.16 (32.35, 57.16)	0.501
TFQI_FT3_	−0.13 (−0.27, 0.01)	−0.18 (−0.32, −0.02)	< 0.001	−0.13 (−0.27, 0.02)	−0.18 (−0.27, −0.03)	0.035
TFQI_FT4_	−0.12 (−0.32, 0.07)	−0.06 (−0.28, 0.12)	0.001	−0.07 (−0.24, 0.09)	−0.004 (−0.21, 0.12)	0.040
INS (uU/mL)	7.18 (4.93, 10.32)	4.74 (3.28, 7.10)	< 0.001	7.02 (4.85, 9.87)	4.97 (3.50, 7.34)	< 0.001
HOMA IR	1.71 (1.12, 2.57)	1.08 (0.70, 1.71)	< 0.001	1.69 (1.12, 2.51)	1.15 (0.77, 1.81)	< 0.001
GLU (mmol/L)	5.17 (4.82, 5.67)	5.00 (4.67, 5.54)	< 0.001	5.24 (4.84, 5.76)	5.14 (4.79, 5.72)	0.268
TG (mmol/L)	1.42 (0.97, 2.07)	1.24 (0.89, 1.70)	< 0.001	1.29 (0.89, 1.97)	1.12 (0.84, 1.65)	0.019
CHOL (mmol/L)	4.70 (4.15, 5.30)	4.69 (4.14, 5.33)	0.684	4.65 (4.07, 5.21)	4.81 (4.16, 5.46)	0.192
HDL (mmol/L)	1.22 (1.04, 1.42)	1.33 (1.14, 1.53)	< 0.001	1.23 (1.04, 1.42)	1.40 (1.22, 1.59)	< 0.001
LDL (mmol/L)	2.70 (2.21, 3.20)	2.68 (2.21, 3.16)	0.755	2.63 (2.18, 3.18)	2.66 (2.24, 3.24)	0.693
Crea (umol/L)	77.90 (71.00, 86.70)	76.60 (68.80, 85.60)	0.007	80.60 (74.20, 89.50)	80.60 (72.30, 88.00)	0.333
eGFR	83.87 (76.08, 93.14)	86.05 (77.55, 95.38)	0.010	82.43 (74.70, 91.10)	83.35 (74.76, 91.55)	0.643
UA (umol/L)	320.30 (273.60, 380.20)	307.80 (260.65, 361.25)	0.001	324.85 (274.90, 385.60)	315.25 (274.05, 375.30)	0.359
AST (U/L)	26 (22, 32)	26 (23, 32)	0.358	26 (22, 32)	26 (22, 30)	0.834
ALT (U/L)	24 (18, 33)	19 (15, 27)	< 0.001	23 (17, 32)	20 (15, 26)	< 0.001
AST/ALT	1.10 (0.88, 1.33)	1.37 (1.08, 1.67)	< 0.001	1.11 (0.88, 1.37)	1.31 (1.09, 1.70)	< 0.001
Vit D (ng/mL)	18.90 (15.20, 23.30)	18.40 (14.60, 22.50)	0.026	19.60 (15.80, 23.90)	18.65 (14.75, 24.05)	0.277
CC: Total	35.40 (33.60, 37.30)	31.60 (30.20, 33.40)	< 0.001	35.60 (33.80, 37.50)	32.00 (30.50, 33.45)	< 0.001
CC: Men	36.00 (34.20, 37.90)	32.65 (31.00, 34.00)	< 0.001	36.00 (34.20, 37.90)	32.40 (30.90, 33.90)	< 0.001
CC: Women	35.00 (33.30, 37.00)	31.00 (29.85, 32.50)	< 0.001	35.50 (33.45, 37.10)	31.50 (30.30, 32.95)	< 0.001

*Note:* ALT, alanine aminotransferase; AST, aspartate aminotransferase; AST/ALT, AST to ALT ratio; CHOL, total cholesterol; CREA, creatinine; FT3, free triiodothyronine; FT4, free tetraiodothyronine; FT3 to FT4 ratio; TG, triglycerides; INS, fasting insulin; TT4RI: T4 resistance index of thyroid‐stimulating hormone; TT3RI: T3 resistance index of thyroid‐stimulating hormone; TFQIFT3, free T3 index based on thyroid feedback quartiles; TFQIFT4, free T4 index based on thyroid feedback quartiles; Vit D, 25‐OH‐Vit D.

Abbreviations: BMI, body mass index; CC, calf circumference; DBP, diastolic blood pressure; GLU, fasting blood glucose; HDL, high‐density lipoprotein; LDL, low‐density lipoprotein; SBP, systolic blood pressure; TSH, thyroid stimulating hormone; TSHI, thyroid hormone sensitivity index; UA, uric acid.

^a^Nonnormally distribution are presented as medians (P25 and P75).

With respect to the demographic and primary medical traits of the exploratory group, the median age was 60 (ranging from 54 to 66) years for the control group and 66 (ranging from 60 to 73) years for the sarcopenia group. The median DBP was 81 (ranging from 74 to 89) mmHg in the control group, whereas it was 78 (ranging from 71 to 87) mmHg in the sarcopenia group. Hyperlipidemia was present in 21.1% of the control group and 12.2% of the sarcopenia group, with 17.7% of the control group and 22.9% of the sarcopenia group being current smokers.

Individuals with sarcopenia had notably lower BMI, FT3, FT3/FT4, TT3RI, TFQIFT3, INS, HOMA‐IR, GLU, TG, Crea, UA, ALT, vit D, and CC, including total CC, CC in men, and CC in women. Conversely, the sarcopenia group had significantly higher levels of FT4, TFQIFT4, HDL, eGFR, and AST/ALT.

In the validation cohort, there were no significant disparities in age, thyroid function parameters, fasting glucose levels, lipid profiles, liver function, or renal function between the participants and those in the exploratory group. Notably, the prevalence of sarcopenia was greater in the exploratory group (18.3%) than that in the validation group (10.7%).

### 3.2. Univariate Logistic Regression Models in the Exploratory Cohort

**FIGURE 1:**
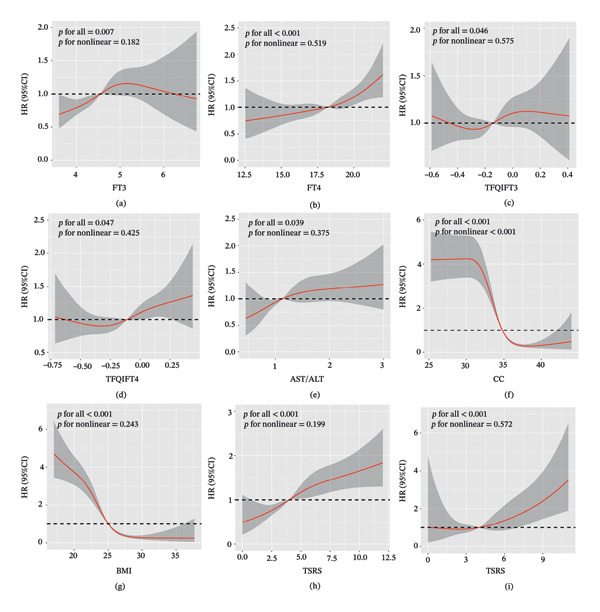
Exploring the nonlinear relationships between various indicators and sarcopenia through restricted cubic spline (RCS) analysis. (a) FT3 and sarcopenia, (b) FT4 and sarcopenia, (c) TFIQ‐FT3 and sarcopenia, (d) TFIQ‐FT4 and sarcopenia, (e) AST/ALT and sarcopenia, (f) CC and sarcopenia, (g) BMI and sarcopenia, (h) TSRS in the exploration cohort and sarcopenia, and (i) TSRS in the validation cohort and sarcopenia.

Analysis with a restricted cubic spline revealed a linear correlation between continuous factors and the probability of having sarcopenia onset (overall, *p*  <  0.05; nonlinearity, *p*  >  0.05), excluding CC (Figure 1). Following the implementation of univariate logistic regression for variable selection, the ultimate logistic model for screening for sarcopenia probability featured 9 distinct variables (Table 2), each of which exhibited statistical significance (*p*  <  0.05). The logistic regression analysis demonstrated that advanced age, male gender, low BMI, reduced CC, decreased FT3, low TFQIFT3, elevated AST/ALT, high FT4, and increased TFQIFT4 correlated significantly with the prevalence of sarcopenia among euthyroid participants. The Hosmer–Lemeshow test validated the model’s goodness of fit, confirming that these univariate models aligned with the actual prevalence rates observed (*p*  >  0.05).

**TABLE 2:** Univariate logistic regression models in the exploratory cohort.

	*β*‐coefficient	S.E	Wald	OR (95% CI)
AGE	0.092	0.006	1.097	1.097 (1.083, 1.111)
Gender	0.193	0.100	3.742	1.213 (1.017, 1.476)
AST/ALT	1.388	0.112	153.234	4.006 (3.216, 4.991)
CC	−0.506	0.025	408.705	0.603 (0.574, 0.633)
BMI	−0.413	0.021	373.919	0.662 (0.635, 0.690)
FT3 (pmol/L)	−0.491	0.180	7.456	0.612 (0.430, 0.871)
FT4 (pmol/L)	0.104	0.023	19.998	1.110 (1.060, 1.161)
TFQI_FT3_	−1.087	0.248	19.291	0.337 (0.207, 0.548)
TFQI_FT4_	0.617	0.194	10.091	1.853 (1.267, 2.712)

### 3.3. LASSO Variable Selection

**FIGURE 2:**
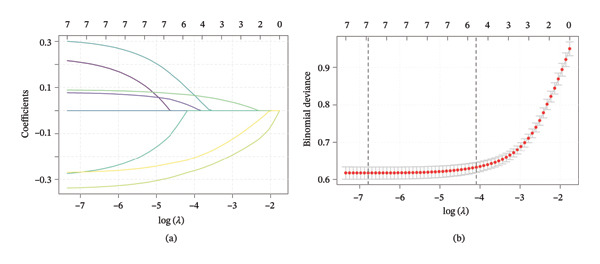
Variable selection using least absolute shrinkage and selection operator (LASSO) regression with 10‐fold cross‐validation. (a) Coefficient profiles of the variables plotted against log (*λ*). Each colored curve represents a variable; the vertical dashed line indicates the optimal *λ* value chosen by the 1‐SE criterion (*λ* = 0.0167), at which five variables had nonzero coefficients. (b) Cross‐validation curve. The *y*‐axis shows binomial deviance; the left‐hand vertical dashed line marks lambda.min, and the right‐hand dashed line marks lambda.1se. The five retained variables were FT4, AST/ALT ratio, age, calf circumference, and BMI.

Of the clinical and laboratory variables, LASSO regression with 10‐fold cross‐validation and the 1‐SE rule selected five variables with nonzero coefficients: FT4, AST/ALT ratio, age, calf circumference, and BMI (Figure 2a). The optimal *λ* value was 0.0167 (Figure 2b).

### 3.4. Multivariate Model for Logistic Regression Analysis in Exploratory Populations

The five LASSO‐selected variables were entered into a multivariable logistic regression model. All five variables remained in the model. Higher FT4 (OR: 1.11, 95% CI: 1.06–1.16, and *p*  <  0.001), higher AST/ALT ratio (OR: 1.29, 95% CI: 0.97–1.71, and *p* = 0.080), and older age (OR: 1.09 per year, 95% CI: 1.08–1.11, and *p*  <  0.001) were positively associated with sarcopenia, whereas larger CC (OR: 0.72 per cm, 95% CI: 0.68–0.77, and *p*  <  0.001) and higher BMI (OR: 0.76 per kg/m^2^, 95% CI: 0.72–0.80, and *p*  <  0.001) were inversely associated with sarcopenia. The regression coefficients and odds ratios are detailed in Table 3. For comparison, the original six‐variable model derived by backward stepwise logistic regression (including gender and TFQIFT4 in place of FT4) is reported in Supporting Table 1 as a sensitivity analysis. Its discriminative performance was slightly lower than that of the five‐variable LASSO‐based score (AUC 0.823 vs. 0.845 in the exploratory cohort).

**TABLE 3:** Multivariable logistic regression model for sarcopenia screening based on LASSO‐selected variables in euthyroid participants.

	*β* coefficient	S.E	OR (95% CI)	*p*
FT4	0.10	0.03	1.11 (1.05–1.17)	< 0.001
AST/ALT	0.25	0.15	1.29 (0.97–1.71)	0.080
AGE	0.09	0.01	1.09 (1.08–1.11)	< 0.001
CC	−0.32	0.03	0.72 (0.68–0.77)	< 0.001
BMI	−0.28	0.03	0.76 (0.72–0.80)	< 0.001

### 3.5. Novel Alternative Screening Score for the Probability of Sarcopenia in Chinese Middle‐Aged and Elderly Individuals With Normal Thyroid Function Using Multivariate Modeling

**FIGURE 3:**
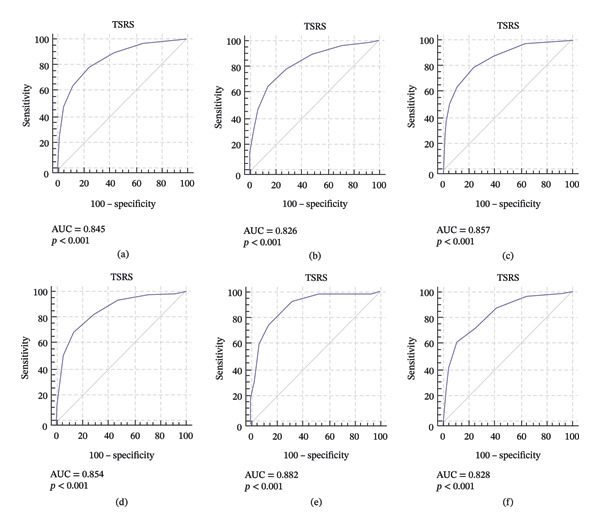
Receiver operating characteristic (ROC) curves displaying the probability of sarcopenia occurrence for all participants in the exploratory cohort ((a) total; (b) males; and (c) females) and validation cohort ((d) total; (e) males; and (f) females) on the basis of their sarcopenia screening scores.

Table 4 illustrates the newly introduced thyroid sarcopenia risk score (TSRS), which serves as an innovative alternative approach for assessing the likelihood of sarcopenia in middle‐aged and older Chinese individuals with normal thyroid hormone function. This novel scoring system, built upon five variables and regression coefficients, spans from 0 to 14. The diagnostic performance of this scoring system is summarized in Table 5. In regard to screening for sarcopenia rates, the optimal cutoff points were 3 for the mixed group, 4 for men, and 3 for women. The Youden’s indices peaked at 0.535, 0.504, and 0.556 for these respective groups. The performance metrics revealed that the approach achieved 78.3% sensitivity and 75.1% specificity across the board, with men having 64.6% sensitivity and 85.7% specificity and women having 78.6% sensitivity and 76.9% specificity. In terms of the predictive value, the mixed cohort had a 18.3% PPV, 20.1% for men and 17.2% for women, all with negative predictive values exceeding 79.9%. ROC curve analyses (Figure 3) in the initial cohort revealed an AUC of 0.845 (95% CI: 0.831–0.859) overall (Figure 3a), which matched that of males (0.826; 95% CI: 0.801–0.848) (Figure 3b), while the performance of women was stronger (0.857; 95% CI: 0.839–0.873) (Figure 3c). The validation cohort continued this impressive run, with AUCs of 0.854 (95% CI: 0.831–0.874) for all participants (Figure 3d), 0.882 (95% CI: 0.852–0.908) for men (Figure 3e), and 0.828 (95% CI: 0.794–0.859) for women (Figure 3f). At the ≥ 4 threshold, the sensitivity and specificity for all individuals in the validation cohort were 68.4% and 87.0%, respectively, with positive and negative predictive values of 10.7% and 89.3%, respectively. When the cutoff was 4, male participants demonstrated a sensitivity and specificity of 75.4% and 84.4%, respectively, yielding positive and negative predictive values of 11.4% and 88.6%, respectively. At the four‐point threshold, female subjects exhibited sensitivity and specificity of 60.7% and 89.4%, respectively, with corresponding positive and negative predictive values of 10.1% and 89.9%, respectively. Based on the new five‐variable screening score, the AUC was 0.845 in the exploratory cohort and 0.854 in the validation cohort. The calibration plot demonstrated good agreement between predicted probabilities and observed sarcopenia prevalence in both cohorts (Supporting Figure 2). The Brier score was 0.092 in the exploratory cohort and 0.068 in the validation cohort, confirming satisfactory calibration. The calibration plots are presented in Supporting Figure 2.

**TABLE 4:** Sarcopenia screening score for probability assessment in middle‐aged and elderly euthyroid participants via logistic regression analysis.

Characteristic	Scores
Age (years)	
50–65	0
65–74	2
≥ 75	3
BMI (kg/m^2^)	
≥ 24	0
18.5–24	1
< 18.5	2
FT4 (pmol/L)	
< 17.0	0
17.0–20.0	1
≥ 20.0	2
AST/ALT	
≥ 1.3	1
< 1.3	0
CC	—
Men	
≥ 34	0
32–34	1
< 32	3
Women	
≥ 33	0
31–33	1
< 31	3
Total possible score	0‐14

**TABLE 5:** Diagnostic accuracy metrics for sarcopenia screening scores in the exploratory and validation cohorts.

	AUC	95% CI	Youden’s index	Cutoff point	Sensitivity (%)	Specificity (%)	Positive predictive value (%)	Negative predictive value (%)
Exploratory subjects								
All	0.845	0.831–0.859	0.535	3	78.3	75.1	18.3	81.7
Men	0.826	0.801–0.848	0.504	4	64.6	85.7	20.1	79.9
Women	0.857	0.839–0.873	0.556	3	78.6	76.9	17.2	82.8
Verification subjects								
All	0.854	0.831–0.874	0.553	4	68.4	87.0	10.7	89.3
Men	0.882	0.852–0.908	0.598	4	75.4	84.4	11.4	88.6
Women	0.828	0.794–0.859	0.500	4	60.7	89.4	10.1	89.9

## 4. Discussion

Sarcopenia, a prevalent skeletal muscle disorder, is characterized by diminished muscle mass and impaired muscle function. Skeletal muscle serves as a crucial target organ for thyroid hormones and is capable of influencing human body composition and movement through mitochondrial T3 receptors [16, 17]. Recently, interest in not only thyroid hormone levels themselves [3, 18] but also reduced sensitivity to these hormones has increased [19]. This phenomenon may explain why individuals with normal thyroid function sometimes exhibit clinical manifestations of hypothyroidism [20]. This study provides further evidence of the role of thyroid hormones, which reduce sensitivity to these hormones, in the screening of sarcopenia among middle‐aged and elderly individuals without apparent thyroid dysfunction. However, previous research has largely focused on exploring the relationships among thyroid hormones, reduced sensitivity, and the onset of sarcopenia. To our knowledge, no studies have yet translated these indicators into clinical screening applications. To bridge this gap and translate our findings into tangible patient benefits, we developed a screening method based on thyroid hormones and their sensitivity to assess the probability of having sarcopenia in middle‐aged and older adults with normal thyroid function. This approach could prove invaluable to the field, particularly in resource‐limited settings where it holds significant clinical utility.

To facilitate clinical implementation, we developed a novel sarcopenia screening score on the basis of our findings, specifically designed for euthyroid individuals with muscle wasting issues. This scoring system incorporates age, BMI, AST/ALT, CC, and FT4 levels. Notably, the LASSO procedure retained FT4—the directly measured free thyroxine concentration—rather than the derived TFQIFT4 index used in our earlier stepwise model (Supporting Table 1). This finding indicates that, among euthyroid individuals, circulating FT4 concentrations alone already capture the thyroid‐related variance relevant to sarcopenia risk. Our research demonstrates that this new risk assessment tool boasts impressive diagnostic accuracy, making it more capable of detecting and screening for sarcopenia in Chinese adults aged 50 and over with normal thyroid function. The ROC curves, which represent our consistency statistics, revealed AUC values of 0.845 (95% CI: 0.831–0.859) for exploratory cohorts and 0.854 (95% CI: 0.831–0.874) for split sample internal validation, highlighting the strong discriminatory power of our new scoring system in terms of identifying at‐risk euthyroid patients. In sensitivity analyses, the six‐variable backward stepwise model that included TFQIFT4 yielded comparable but slightly inferior discrimination (Supporting 2), supporting the robustness of the parsimonious LASSO‐derived five‐variable model. Furthermore, the low Brier scores and well‐calibrated plots confirmed that this sarcopenia screening score achieves remarkable precision in distinguishing between patients with and without muscle wasting. Calibration of the screening score was rigorously evaluated. The calibration plots showed close alignment between predicted and observed probabilities, and the low Brier scores (0.092 and 0.068 in the exploratory and validation cohorts, respectively) confirm that the score accurately estimates the probability of having sarcopenia.

Mounting evidence suggests a positive correlation between reduced thyroid hormone sensitivity and metabolic disorders, including diabetes, metabolic syndrome, and frailty [13, 14, 21–24]. In our initial stepwise model, TFQIFT4 was significantly associated with sarcopenia. However, when more rigorous LASSO regularization was applied, TFQIFT4 was not retained; instead, FT4 itself was selected. This suggests that, in euthyroid middle‐aged and elderly individuals, the directly measured FT4 concentration already captures the relevant thyroid‐related variance for sarcopenia screening. From a practical standpoint, using a single routine biochemical marker rather than a derived index simplifies the screening tool and facilitates its implementation in resource‐limited settings. Similarly, recent evidence has indicated that peripheral thyroid hormone sensitivity mediates the link between body composition and diabetes [25], and central sensitivity indices are associated with metabolic syndrome components in Chinese euthyroid adults [26].

Our research focused on participants who were fifty years of age or older. The literature indicates that muscle mass and strength typically start to decline noticeably around the fifties, accelerating after the sixtieth birthday, and men appear to be particularly vulnerable [27]. Consequently, the AWGS recommends screening for sarcopenia among community‐dwelling seniors and those with specific underlying conditions, both of which are associated with heightened risk [27]. These findings suggest that middle to late adulthood presents a prime window for detecting sarcopenia, enabling timely intervention to stabilize or reverse the condition before it leads to unfavorable health outcomes. In accordance with the AWGS 2019 guidelines, the recommended CC cutoff is under 34 cm for men and under 33 cm for women [28]. AST/ALT reportedly predicts muscle mass reduction [29], corroborating our findings.

This pioneering exploratory study seeks to develop a straightforward, user‐friendly screening tool for detecting sarcopenia among middle‐aged and elderly individuals with normal thyroid function. While the current gold standard diagnosis relies on the EWGSOP [4] or AWGS [28] criteria, these approaches have a fair share of their limitations. Diagnostic outcomes often vary depending on the equipment used, require considerable time, and may be subject to bias because of racial diversity in cutoff values along with differences in body shape and size. Many healthcare facilities in our country lack body composition analyzers, but the radiation exposure associated with DXA and the steep costs of computed tomography and magnetic resonance imaging restrict their widespread clinical application. For these reasons, our novel alternative sarcopenia screening score is effective when body composition assessment is not available—boasting no radiation exposure and serving as a more practical and budget‐friendly option than CT, MRI, or DXA. The parameters needed to calculate this new score are a piece of cake to obtain: BMI can be calculated from height and weight measurements; CC is measured with a simple tape measure; and the other indicators are standard laboratory tests including AST, ALT, TSH, and FT4. Catching sarcopenia early using readily available conventional metrics can help prevent further physical disability by offering these patients a fighting chance to restore muscle mass and function. Overall, our alternative sarcopenia screening score represents an effective and convenient health promotion tool rather than a diagnostic test, making it an excellent alternative for screening community‐dwelling middle‐aged and older adults with normal thyroid function for sarcopenia.

A major strength of this research lies in its comprehensive coverage of middle‐aged and elderly individuals from China’s western regions. However, the study does have several limitations that must be addressed. First, the relatively low prevalence of sarcopenia in our current investigation resulted in a relatively small sample size of sarcopenia patients, which could negatively impact the statistical power. Future research will need to expand the sample size, and given China’s vast population diversity, further validation of screening scores is necessary for other regions across the country. Additionally, our analysis specifically targeted middle‐aged and elderly Chinese individuals with normal thyroid function. Consequently, our findings may not be generalizable to populations with thyroid dysfunction or to other racial/ethnic groups or individuals from different countries. Subsequent studies will aim to test the performance and applicability of these tools across various populations and settings. Third, individuals who did not undergo sarcopenia screening or whose data were incomplete were excluded from our analysis, potentially affecting the final dataset. Fourth, although our model was validated in an independent split‐sample cohort, true external validation in geographically and temporally distinct populations was not performed. This limits generalizability, and future studies should conduct prospective external validation. Nevertheless, the large‐scale, multicenter design of this study lends considerable credibility to our findings, and despite these limitations, the results remain highly reliable. This research sought to develop a screening tool for sarcopenia in Chinese individuals with normal thyroid function that could be effectively implemented in clinical practice.

In summary, researchers have crafted a novel alternative risk assessment score for sarcopenia based on thyroid function parameters to detect this condition among individuals with normal thyroid function. This score has demonstrated both high internal consistency and remarkable accuracy. The newly developed assessment score is an effective screening tool that is not only cost‐effective and straightforward to implement but also particularly valuable in resource‐limited settings. Furthermore, it has been validated for identifying sarcopenia in middle‐aged and elderly populations—groups that represent prime candidates for intervention programs aimed at preventing further physical disability in China’s clinical practice.

## Author Contributions

Fei Ding: played a key role in developing the study’s conceptual framework, curated and analyzed the data, and took the lead in drafting the initial manuscript while also providing critical revisions throughout the writing process. Jirong Yue: helped shape the study’s conceptual approach and offered insightful edits to improve the final version. Yong He: spearheaded the conceptual development, secured necessary funding, conducted investigations, established the methodology, oversaw project administration, provided supervision, and reviewed the manuscript for publication.

Guarantor: Yong He.

## Funding

This research was funded by China’s National Key R&D Program (2020YFC2005600) and the Sichuan Provincial Science and Technology Department (Grant no. 2024YFFK0144).

## Disclosure

The manuscript has been read and approved by all the authors so that the requirements for authorship as stated earlier in this document have been met and that each author believes that the manuscript represents honest work if that information is not provided in another form.

## Ethics Statement

The research protocol received formal endorsement from the institutional review board at West China Hospital, Sichuan University (approval ID: 2017‐445), with all procedures being conducted in strict compliance with the established ethical standards.

## Conflicts of Interest

The authors declare no conflicts of interest.

## Supporting Information

Additional supporting information can be found online in the Supporting Information section.

## Supporting information


**Supporting Information 1** Supporting Figure 1: Flowchart of the study subject screening process.

**Supporting Information 2** Supporting Table 1: Sensitivity analysis: odds ratios (95% CI) and β coefficients for sarcopenia prevalence in the euthyroid population using backward stepwise likelihood ratio multivariate logistic regression (original six‐variable model).

**Supporting Information 3** Supporting Figure 2: Calibration plots of the sarcopenia screening score in the exploratory cohort (A) and the validation cohort (B). The 45° dashed line denotes perfect calibration; the solid and dotted curves show logistic and nonparametric calibration, respectively. The Brier score was 0.092 in the exploratory cohort and 0.068 in the validation cohort, both well below 0.25, indicating satisfactory calibration. Key calibration statistics are reported in each panel.

## Data Availability

Data used to support the findings of this study are available on request from the corresponding authors.
