# Supplementary material for: A Novel Sarcopenia Screening Score Based on Thyroid Function Parameters in Euthyroid Middle‐Aged and Elderly Chinese Adults
Source: Int J Endocrinol. 2026 Jun 24;2026:1577695. doi: 10.1155/ije/1577695 (PMC13291889; doi:10.1155/ije/1577695)
Supplement: Supplementary file 1 — Supporting Information 1 Supporting Figure 1: Flowchart of the study subject screening process. [file IJE-2026-1577695-s002.pptx]

## Slide 1
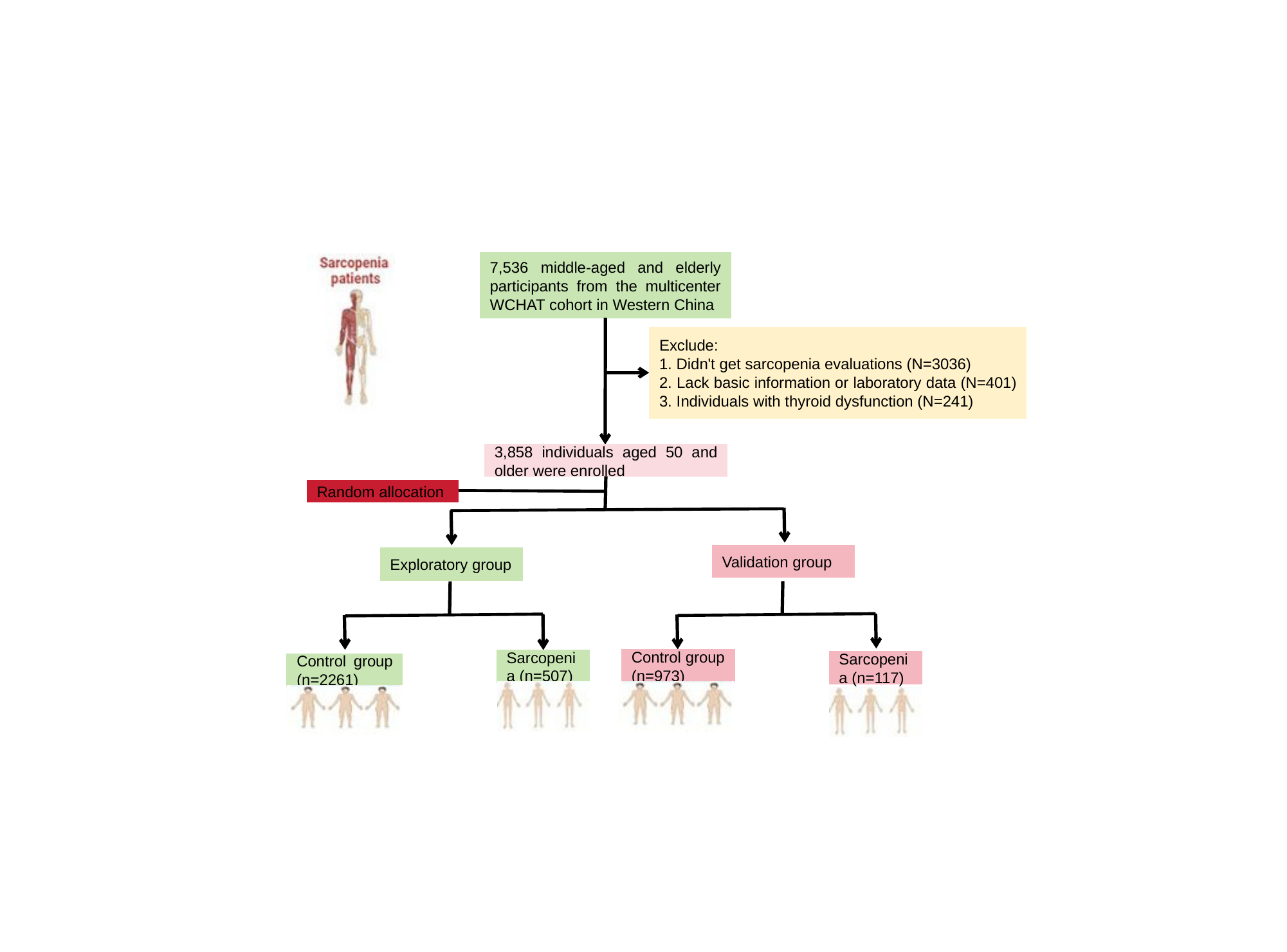

7,536 middle-aged and elderly participants from the multicenter WCHAT cohort in Western China
Exclude:
1. Didn't get sarcopenia evaluations (N=3036)
2. Lack basic information or laboratory data (N=401)
3. Individuals with thyroid dysfunction (N=241)
3,858 individuals aged 50 and older were enrolled
Random allocation
Validation group
Exploratory group
Control group (n=973)
Sarcopenia (n=507)
Sarcopenia (n=117)
Control group (n=2261)
