# Supplementary figures and images for: A Novel Sarcopenia Screening Score Based on Thyroid Function Parameters in Euthyroid Middle‐Aged and Elderly Chinese Adults
Source: Int J Endocrinol. 2026 Jun 24;2026:1577695. doi: 10.1155/ije/1577695 (PMC13291889; doi:10.1155/ije/1577695)

## Slide 1
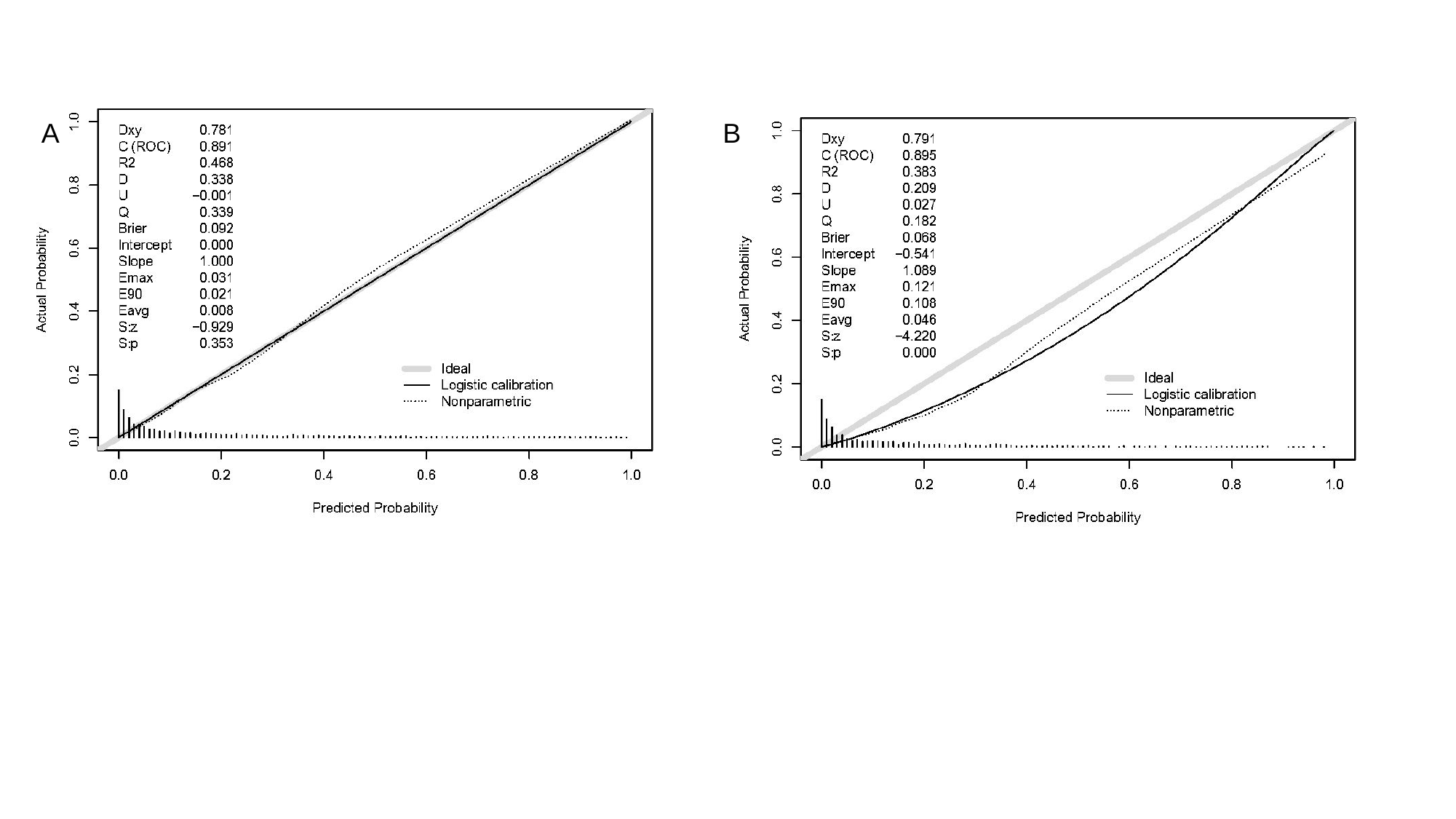

A
B

Supplement: Supplementary file 2 — Supporting Information 2 Supporting Table 1: Sensitivity analysis: odds ratios (95% CI) and β coefficients for sarcopenia prevalence in the euthyroid population using backward stepwise likelihood ratio multivariate logistic regression (original six‐variable model). [file IJE-2026-1577695-s003.pptx]
