# Supplementary material for: A Novel Sarcopenia Screening Score Based on Thyroid Function Parameters in Euthyroid Middle‐Aged and Elderly Chinese Adults
Source: Int J Endocrinol. 2026 Jun 24;2026:1577695. doi: 10.1155/ije/1577695 (PMC13291889; doi:10.1155/ije/1577695)
Supplement: Supplementary file 3 — Supporting Information 3 Supporting Figure 2: Calibration plots of the sarcopenia screening score in the exploratory cohort (A) and the validation cohort (B). The 45° dashed line denotes perfect calibration; the solid and dotted curves show logistic and nonparametric calibration, respectively. The Brier score was 0.092 in the exploratory cohort and 0.068 in the validation cohort, both well below 0.25, indicating satisfactory calibration. Key calibration statistics are reported in each panel. [file IJE-2026-1577695-s001.docx]

**Supplementary Table 1** Sensitivity analysis: odds ratios (95% CI) and β coefficients for sarcopenia prevalence in the euthyroid population using backward stepwise likelihood ratio multivariate logistic regression (original six‑variable model)

|  | β Coefficient | S.E | OR (95% CI) |
| --- | --- | --- | --- |
| Gender | 0.523 | 0.205 | 1.686 (1.128, 2.521) |
| TFQIFT4 | 0.659 | 0.247 | 1.933 (1.192, 3.134) |
| AST/ALT | 0.270 | 0.146 | 1.311 (1.014, 1.746) |
| AGE | 0.086 | 0.008 | 1.089 (1.072, 1.107) |
| CC | -0.348 | 0.031 | 0.706 (0.665, 0.749) |
| BMI | -0.263 | 0.028 | 0.768 (0.728, 0.811) |

Using backwards stepwise likelihood ratio multivariate logistic regression.
